# The RecQ DNA helicase Rqh1 constrains Exonuclease 1-dependent recombination at stalled replication forks

**DOI:** 10.1038/srep22837

**Published:** 2016-03-09

**Authors:** Fekret Osman, Jong Sook Ahn, Alexander Lorenz, Matthew C. Whitby

**Affiliations:** 1 Department of Biochemistry, University of Oxford, South Parks Road, Oxford, OX1 3QU UK

## Abstract

DNA double-strand break (DSB) repair by homologous recombination (HR) involves resection of the break to expose a 3′ single-stranded DNA tail. In budding yeast, resection occurs in two steps: initial short-range resection, performed by Mre11-Rad50-Xrs2 and Sae2; and long-range resection catalysed by either Exo1 or Sgs1-Dna2. Here we use genetic assays to investigate the importance of Exo1 and the Sgs1 homologue Rqh1 for DNA repair and promotion of direct repeat recombination in the fission yeast *Schizosaccharomyces pombe*. We find that Exo1 and Rqh1 function in alternative redundant pathways for promoting survival following replication fork breakage. Exo1 promotes replication fork barrier-induced direct repeat recombination but intriguingly limits recombination induced by fork breakage. Direct repeat recombination induced by ultraviolet light depends on either Exo1 or Rqh1. Finally, we show that Rqh1 plays a major role in limiting Exo1-dependent direct repeat recombination induced by replication fork stalling but only a minor role in constraining recombination induced by fork breakage. The implications of our findings are discussed in the context of the benefits that long-range resection may bring to processing perturbed replication forks.

Homologous recombination (HR) is a central mechanism for the repair of DNA double-strand breaks (DSBs)^1^, and also plays a key role in processing perturbed replication forks, including promoting the restart of blocked and broken forks^2^. It is typically initiated from an exposed DNA end (e.g. a DSB or an extruded DNA arm at a reversed replication fork) that has been resected to form a 3′ single-strand tail. This tail acts as a nucleation site for the Rad51 recombinase, which then uses the tail to search for and invade an intact homologous duplex sequence. Strand invasion forms a displacement (D) loop, with the end of the invading strand acting as a primer for a DNA polymerase. From this point HR can progress via several different paths depending on the context of the initiating lesion/perturbed replication fork. In the case of DSBs in G2-phase cells, the D-loop would typically be extended by the polymerase catalysing new DNA synthesis and then unwound by a DNA helicase so the extended strand can anneal to the other side of the break. This mode of DSB repair is called Synthesis-Dependent Strand Annealing (SDSA)^3^. If the D-loop is not unwound, then the single-strand on the other end of the break can anneal to it, ultimately leading to the covalent attachment of the two recombining DNAs via a double Holliday junction. This structure is later resolved so that the DNAs can segregate during mitosis/meiosis^1^,^3^. DSB resection can also promote a Rad51-independent mode of repair called single-strand annealing (SSA), which involves base-pairing between repetitive sequences that flank the break, resulting in the deletion of one of the repeats, as well as the DNA between them^3^.

DNA resection is the key step that assigns repair to HR rather than the alternative mechanism of non-homologous end-joining, which is inherently more mutagenic^4^,^5^. In budding yeast the Mre11-Rad50-Xrs2 (MRX) complex, in conjunction with Sae2, catalyses initial “short-range” DNA resection by cutting the 5′-ended strand up to ~300 nucleotides away from the DNA end, and then degrading it from its point of incision in a 3′ to 5′ direction^6^,^7^. This mode of resection not only generates a short 3′ tail but also removes any protein blocks from the DNA end that could impede exonucleases^8^. The homologous proteins in fission yeast and human, Mre11-Rad50-Nbs1 (MRN) and Ctp1/CtIP, are thought to catalyse short-range resection in a similar manner^7^,^9^,^10^.

Whilst the short 3′ single-strand tails generated by MRX and Sae2 can be sufficient to promote DSB repair, in most instances resection is continued by either of two alternative pathways that generate much longer tails (typically ~2000–6000 nucleotides, but in some instances >50,000 nucleotides)^10^,^11^,^12^,^13^,^14^,^15^. The first pathway depends on Exonuclease 1 (Exo1), which degrades the 5′ ended strand within duplex DNA to generate the requisite 3′ tail^16^. The second pathway utilises the single-strand specific exonuclease activity of Dna2, together with a RecQ-type DNA helicase (Sgs1 in budding yeast; BLM and/or WRN in human)^17^,^18^,^19^. The “long-range” resection catalysed by these pathways is not strictly needed for Rad51-mediated HR, albeit it does improve DSB repair efficiency by 30–50% in budding yeast vegetative cells, leading to speculation as to what its true importance is^11^,^12^,^13^.

Analysis of DSB processing in fission yeast has shown that Exo1 is the primary factor needed for long-range DNA resection in this organism, with the RecQ-type helicase Rqh1 being required for the residual levels of resection in an *exo1*∆ mutant^10^. However, the importance of long-range resection for processing DSBs and perturbed replication forks in fission yeast has not been assessed, and therefore we have conducted a genetic analysis of *exo1* and *rqh1* single and double mutants, measuring genotoxin sensitivity, meiotic recombination, and direct repeat recombination induced by different agents. Both Exo1 and Rqh1 have roles in addition to catalysing long-range resection, for example Exo1 can promote DNA mismatch repair by excising the daughter strand following recognition of the mispaired bases^20^,^21^, and Rqh1 promotes crossover avoidance during DSB repair^22^, most likely by catalysing double Holliday junction dissolution^23^. These additional functions complicate the interpretation of data obtained from genetic assays, limiting what one can conclude about the role of long-range resection. Nevertheless, as an *exo1*∆ *rqh1*∆ double mutant exhibits little or no long-range resection^10^, an absence of an additive or synergistic phenotype for this double mutant would suggest that long-range resection is not essential for the process under investigation, whereas a synergistic interaction would suggest an instance where it might be important. With this logic in mind, we present data that suggest an important role for long-range resection, both in the repair of broken replication forks, and in influencing direct repeat recombination at stalled forks.

## Results

### Cells lacking both Exo1 and Rqh1 exhibit a synergistic increase in sensitivity to camptothecin but not to ionising radiation

**Figure 1:**
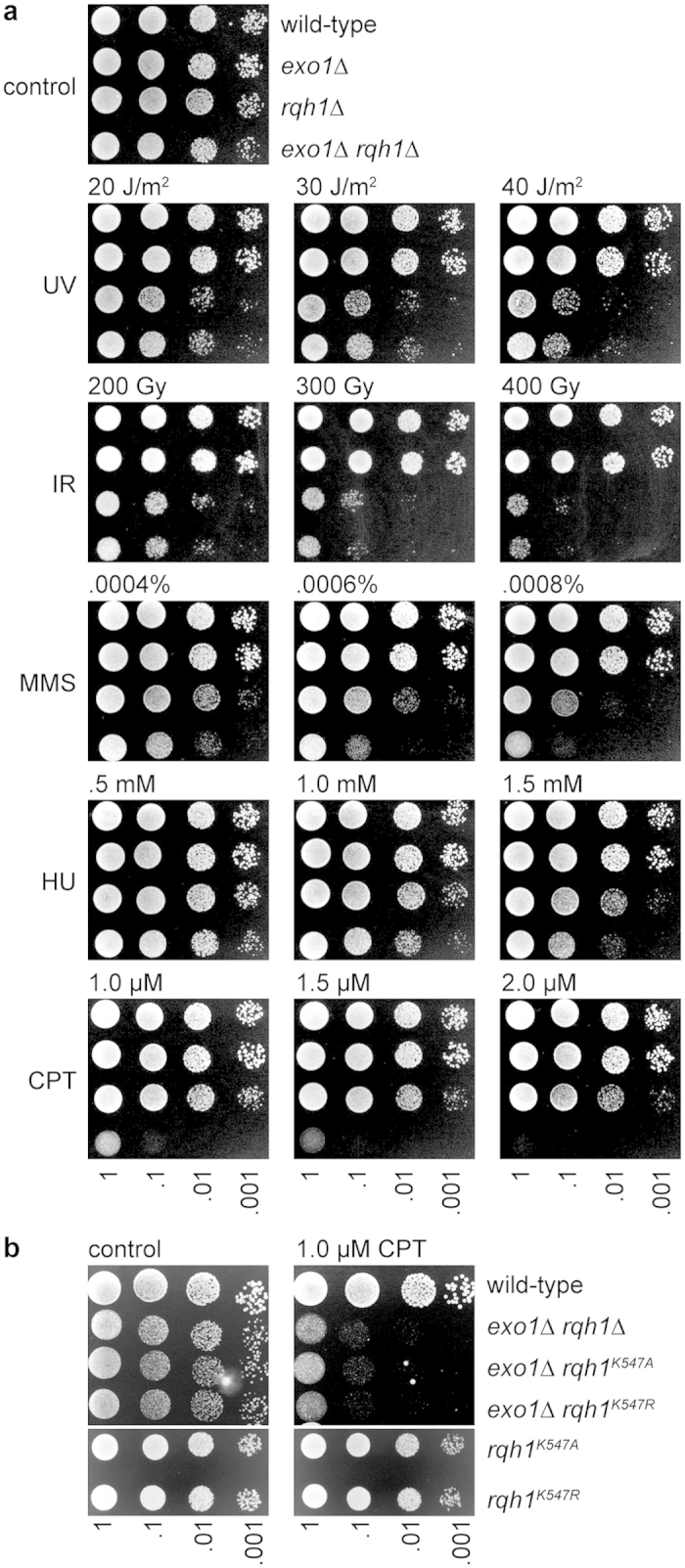
An *exo1*∆ *rqh1*∆ double mutant exhibits a synergistic increase in sensitivity to MMS, HU and CPT. **(a)** Spot assay comparing wild-type (FO656), *exo1*∆ (FO951), *rqh1*∆ (MCW1019) and *exo1*∆ *rqh1*∆ (FO1011) strains for sensitivity to a range of genotoxins. **(b)** Spot assay comparing wild-type (FO656), *exo1*∆ *rqh1*∆ (FO1011), *exo1*∆ *rqh1*^*K547A*^ (MCW4029), *exo1*∆ *rqh1*^*K547R*^ (MCW4030), *rqh1*^*K547A*^ (MCW2453) and *rqh1*^*K547R*^ (MCW2454) strains for sensitivity to CPT.

Previous studies in budding yeast have shown that an *exo1*∆ *sgs1*∆ double mutant exhibits a synergistic increase in sensitivity to a range of DNA damaging agents, including ionising radiation (IR), the IR-mimetic phleomycin, the ribonucleotide reductase inhibitor hydroxyurea (HU), the alkylating agent methyl methanesulphonate (MMS) and the topoisomerase I (Top1) poison camptothecin (CPT)^12^,^15^. Each of these agents can give rise to DSBs either directly, or as a consequence of perturbing DNA replication. Co-depletion of BLM and EXO1 in human cells has also been shown to cause a synergistic increase in CPT sensitivity^15^. To establish whether the same interaction is conserved in fission yeast, we compared the genotoxin sensitivities of *exo1*∆ and *rqh1*∆ single and double mutants (Fig. 1a). Consistent with data from budding yeast and human cells, we found that the fission yeast double mutant exhibits a synergistic increase in sensitivity to MMS, HU and CPT (Fig. 1a). A synergistic increase in sensitivity to CPT was also noted for *exo1*∆, when combined with either *rqh1*^*K547A*^ or *rqh1*^*K547R*^ mutant alleles, which express helicase deficient Rqh1 (Fig. 1b)^24^. However, in contrast to budding yeast, an *exo1*∆ *rqh1*∆ double mutant is no more sensitive to IR than a *rqh1*∆ single mutant. This lack of synergism was also observed for sensitivity to ultraviolet light (UV).

**Figure 2:**
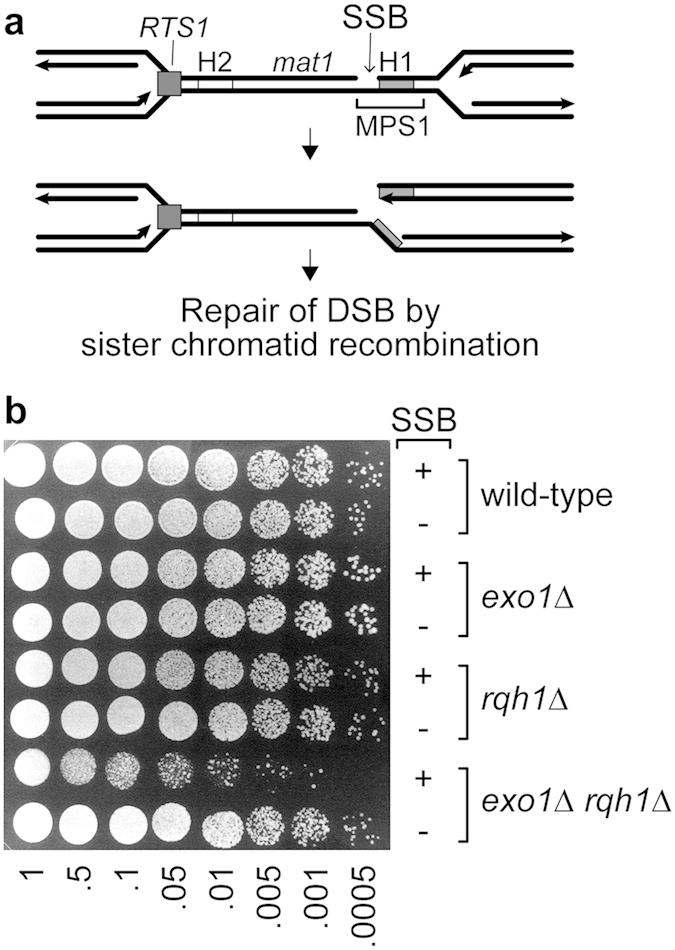
An *exo1*∆ *rqh1*∆ double mutant exhibits a synergistic reduction in growth when SSB formation is active at the *mat1* locus in strains lacking the donor cassettes *mat2*P and *mat3*M. **(a)** Schematic showing the position of the SSB formed within the *mat1* locus and how this is converted into a one-ended DSB during DNA replication. H1 and H2 are the regions of *mat1* that are homologous to both silent mating type donor cassettes *mat2*P and *mat3*M. The replication fork barrier *RTS1* maintains polarity of replication at *mat1*. **(b)** Spot assay comparing the growth of strains MCW3664, MCW3667, MCW3665, MCW3668, MCW3666, MCW3669, MCW3605 and MCW3670. Strains that form a SSB at the *mat1* locus are indicated by a ‘+’ and those that do not by a ‘−’. Cells were diluted in alternating steps of 2- and 5-fold between spots. The plate was incubated at 30 °C for four days before being photographed.

The very strong synergistic interaction between *exo1*∆ and *rqh1*∆ for CPT sensitivity suggests that long-range resection is important for the repair of replication-associated DSBs. CPT impedes the religation step in Top1’s reaction cycle, resulting in an accumulation of single-strand DNA breaks (SSBs), with Top1 attached covalently to the 3′ end of the broken strand. These breaks can be converted into DSBs when encountered by a replication fork^25^. To see whether the requirement for either Exo1 or Rqh1 to repair such lesions was due to the presence of the covalently attached Top1, we made use of strains in which a site-specific SSB without any covalent protein attachment is formed at the fission yeast mating type (MT) locus. Similar to SSBs induced by CPT, the MT locus SSB gives rise to a DSB following its encounter by a replication fork^26^. In a wild-type this DSB is repaired by recombination using one of two homologous silent donor alleles (*mat2*P and *mat3*M), which results in MT switching. However, in the absence of the donors, repair depends on sister chromatid recombination, as it would for the repair of a CPT-induced DSB (Fig. 2a). The data in Fig. 2b show that in the presence of the MT locus SSB, and absence of silent donors, an *exo1*∆ *rqh1*∆ double mutant exhibits reduced viability compared to both wild-type and single mutant strains. These data provide further evidence that DSB resection, catalysed by either Exo1 or Rqh1, may generally be required for the repair of broken replication forks.

### Neither Rqh1 nor Exo1 are essential for meiotic DSB repair in fission yeast

**Figure 3:**
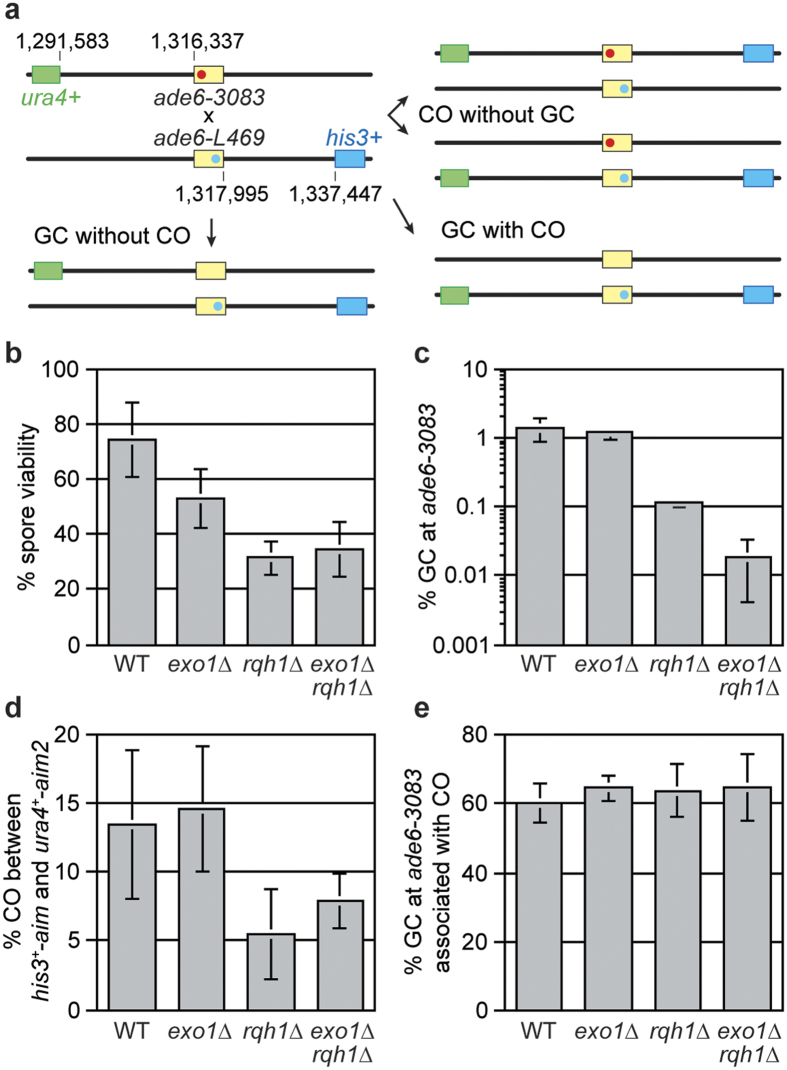
Spore viability and meiotic recombination in *exo1*∆ and *rqh1*∆ single and double mutants. **(a)** Schematic showing the meiotic recombination assay at *ade6* (yellow) and its common outcomes. The positions of *ade6* and the artificially introduced markers *ura4*^+^*-aim2* (green) and *his3*^+^*-aim* (blue) on chromosome 3 are indicated (in bp). The point mutation in *ade6-3083* is shown in red, and *ade6-L469* labelled in light blue. The *ade6-3083* allele is a strong hotspot for meiotic DSB formation, whereas *ade6-L469* is a non-hotspot, and therefore the former acts as the recipient of genetic information in crosses. (**b**) Viability of progeny from wild type and mutant crosses; ALP649xALP688 (WT, n = 10), MCW3748xMCW3749 (*exo1*∆, n = 10), MCW3387xMCW3388 (*rqh1*∆, n = 10), MCW4479xMCW4480 (*exo1*∆ *rqh1*∆, n = 10). (**c**) Frequency of Ade^+^ gene conversions in wild type and mutant crosses; MCW3202xMCW3200 (WT, n = 21), MCW4268xMCW4269 (*exo1*∆, n = 11), MCW3385xMCW3384 (*rqh1*∆, n = 10), MCW4270xMCW4271 (*exo1*∆ *rqh1*∆, n = 13). (**d**) Frequency of crossovers between *ura4*^+^*-aim2* and *his3*^+^*-aim* in wild type and mutant meioses; crosses as in (**c**). (**e**) Frequency of gene conversions associated with a crossover in wild type and mutant crosses; crosses as in (**c**). Data are represented as mean ± standard deviation and n indicates the number of independent crosses.

In budding yeast long-range resection is not needed for efficient formation of D-loops and double Holliday junctions during meiotic DSB repair^11^. To investigate whether it is needed for the recombinational repair of meiotic DSBs in fission yeast, we used a genetic recombination assay containing intragenic markers (*ade6-3083* and *ade6-L469*), and flanking intergenic markers (*his3*^+^*-aim* and *ura4*^+^*-aim2*), to determine gene conversion (GC) and crossover (CO) frequencies, as well as the percentage of GCs at *ade6-3083* associated with a CO^27^,^28^,^29^ (Fig. 3a). To assess the overall efficiency of meiotic DSB repair, we first compared the viability of spores generated from wild-type, *exo1*∆, *rqh1*∆ and *exo1*∆ *rqh1*∆ homozygous crosses (Fig. 3b). Both *exo1*∆ and *rqh1*∆ single mutants exhibit reductions in spore viability compared to wild type, but there is no further decrease in the double mutant. Assuming there is no other enzyme capable of catalysing long-range resection during meiosis in fission yeast, these data suggest that long-range resection is not essential for meiotic DSB repair, albeit it might promote its efficiency. The *exo1*∆ single mutant exhibits the same frequencies of GC and CO as wild-type, and the percentage of GCs associated with a CO is also unchanged (Fig. 3c–e). In contrast, a *rqh1*∆ mutant exhibits significant reductions in both GC (~12-fold, *P* = 4.7 × 10^−10^) and CO (~2.5-fold, *P* = 1.6 × 10^−4^), whilst the percentage of GCs associated with a CO is unchanged (Fig. 3c–e). We suspect that these reductions reflect a loss of Holliday junction branch migration activity, rather than less DNA resection^29^,^30^. The *exo1*∆ *rqh1*∆ double mutant exhibits essentially the same CO frequency as a *rqh1*∆ single mutant (*P* > 0.05), and, as with both single mutants, shows no change in the percentage of GCs associated with a CO (Fig. 3d,e). However, its frequency of GC is ~6-fold lower than a *rqh1*∆ single mutant (*P* = 1.1 × 10^−10^) (Fig. 3c). Altogether these data suggest that the resection activities of Exo1 and Rqh1, whilst not essential for meiotic DSB repair, might be required for efficient GC in fission yeast, possibly by increasing the frequency of interhomolog recombination relative to intersister recombination.

### Replication-associated DSBs are stabilised in an *exo1*∆ *rqh1*∆ double mutant

**Figure 4:**
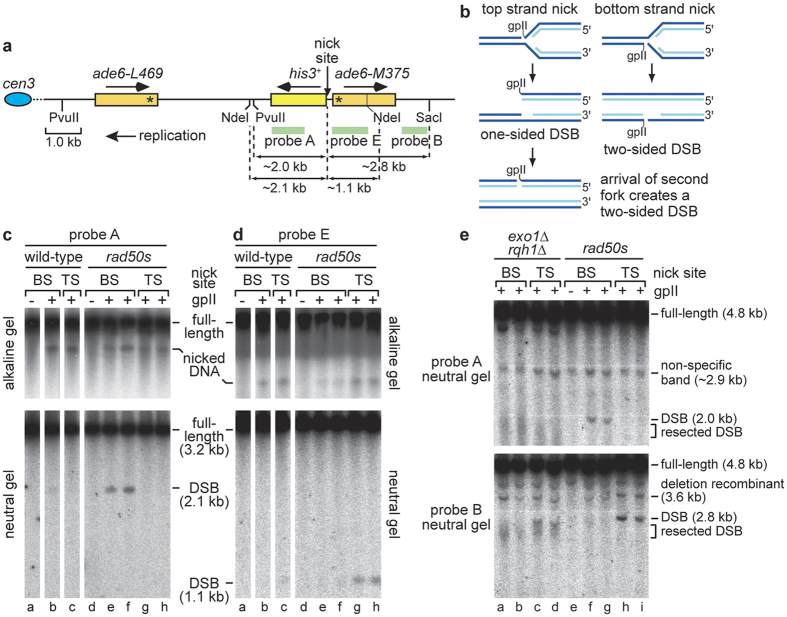
Replication fork breakage at a site-specific SSB. **(a)** Schematic of the *ade6*^−^ direct repeat on chromosome 3 showing the position of the gpII nick site, probes, and relevant restriction sites and fragment sizes. Asterisks indicate the position of the point mutations in *ade6-L469* and *ade6-M375*, and arrows show the direction of replication and transcription as indicated. The distance between the *ade6*^−^ heteroalleles is ~5 kb. **(b)** Model for how replication forks are thought to break at SSBs in the leading or lagging template strand. **(c)** Alkaline and neutral gel analysis of genomic DNA from wild-type and *rad50S* strains containing either a bottom strand (BS) or top strand (TS) nick site, and either pREP1 or pREP1-NLS-gpII. DNA was digested with NdeI and detected with Probe A. For the *rad50S* strains, analysis of genomic DNA from two independent pREP1-NLS-gpII transformants is shown. **(d)** The same as (**c**) except Probe E is used instead of Probe A. **(e)** Neutral gel analysis of genomic DNA from *exo1*∆ *rqh1*∆ and *rad50S* strains containing either a BS or TS nick site, and either pREP1 or pREP1-NLS-gpII as indicated. DNA was digested with PvuII and SacI and detected with Probe A and B as indicated.

To further study what impact the loss of both Exo1 and Rqh1 has on the recombinational repair of broken replication forks, we established a system for measuring recombination caused by an inducible site-specific SSB at the *ade6* locus on chromosome 3 of *S. pombe*. M13 filamentous bacteriophage gene II protein (gpII) was used to make a nick in the DNA between a direct repeat of *ade6*^−^ heteroalleles (Fig. 4a). gpII is a site-specific DNA nicking enzyme that has previously been used for studying replication fork breakage on phage λ chromosomes in *Escherichia coli*^31^. Upon nicking the DNA it forms a covalent complex with the 5′ end of the nick^32^. The minimal nicking site (40 bp) for gpII was inserted between the *ade6*^−^ direct repeats (Fig. 4a). Previous studies, using the polar replication fork barrier *RTS1* inserted at this site, have shown that replication of this region occurs almost exclusively in the telomere to centromere direction^33^,^34^,^35^. Two strains were therefore constructed, one for each orientation of the nicking site, so that we could assess the effect of a nick in both the leading and lagging template strands.

In theory, a nick in the leading template strand (top strand) initially gives rise to a one-sided DSB upon encounter by a replication fork, which can be converted into a two-sided DSB during fork convergence, whereas a lagging strand (bottom strand) nick causes a two-sided DSB following encounter by the first fork (Fig. 4b)^36^. To determine whether a chromosomal nick does indeed give rise to such DSBs, we expressed gpII, with an N-terminal SV40 T-antigen nuclear localisation signal, from the pREP1 thiamine repressible *nmt* promoter, in strains containing the nicking site, and analysed nick and DSB formation on both alkaline and neutral gels (Fig. 4c,d). In wild-type strains, nicking was clearly detected on alkaline gels for both orientations of the cleavage site (Fig. 4c,d top panels, lanes b and c). In contrast only very faint signals for DSBs were detected on neutral gels (Fig. 4c,d bottom panels, lanes b and c). To see whether the failure to clearly detect DSBs was due to the rapidity of their repair, we repeated our experiments in a *rad50S* mutant, which is defective for processing DSBs with covalently attached protein^9^. In this mutant, bands indicative of DSBs were clearly detected (Fig. 4c,d, lanes e–h). Furthermore, by synchronizing cells, using the *cdc10-V50* thermosensitive mutation, we were able to confirm that DSB formation depended on passage through S-phase (see Supplementary Fig. S1). However, whereas we could detect nicked DNA with probes either side of the nicking site (Fig. 4c,d top panels, lanes e–h), we could only clearly detect DSBs in strains with a bottom strand nick using probe A (Fig. 4c bottom panel, lanes e and f) and in strains with a top strand nick using probe E (Fig. 4d bottom panel, lanes g and h). In other words, only the side of the DSB containing the covalently attached gpII is clearly detected, which is consistent with the known deficiency of a *rad50S* mutant in processing “dirty” DNA ends^9^.

Having confirmed that our gpII-based system generates nicks that result in DSBs during S-phase, we next assessed whether deletion of *exo1* and/or *rqh1* resulted in DSB stabilisation. As with wild-type, only faint DSB signals were detected for both single mutants (data not shown), however clear DSB signals were detected in *exo1*∆ *rqh1*∆ double mutant strains with either top or bottom strand nicks, albeit, unlike in a *rad50S* mutant, the signals are smeared (Fig. 4e top and bottom panels, lanes a–d). A similar effect has been seen in a budding yeast *exo1*∆ *sgs1*∆ double mutant and likely reflects the fact that short-range resection, dependent on MRN and Ctp1, is still active in these mutants^14^. Also, unlike a *rad50S* mutant, both ends of the DSB are detected, which is consistent with the expected attenuation of 5′- 3′ resection (Fig. 4e, compare lanes a–d with f–i). Altogether these data show that efficient long-range resection of replication-associated DSBs relies on either Exo1 or Rqh1.

### GpII-induced nicks stimulate direct repeat recombination

**Figure 5:**
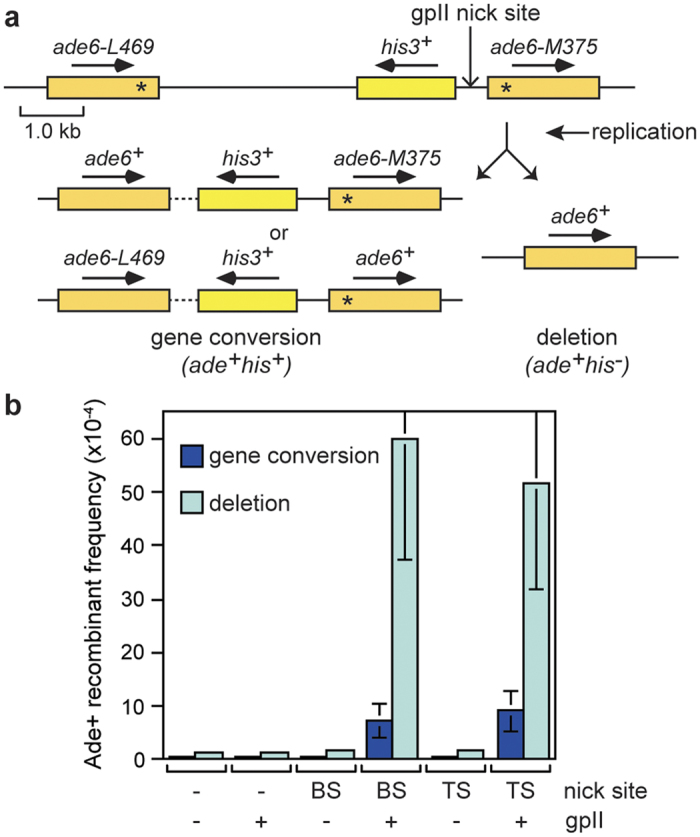
GpII-induced direct repeat recombination in wild-type fission yeast. **(a)** Schematic of the *ade6*^−^ direct repeat on chromosome 3 showing the two types of *ade6*^+^ recombinant. The distance between the *ade6*^−^ heteroalleles is ~5 kb. **(b)** Ade^+^ recombinant frequencies for strains MCW39, MCW1159, and JSA267 carrying either pREP81 (− gpII) or pREP81-NLS-gpII (+ gpII) (see also Supplementary Table S1). BS and TS = bottom and top strand nick site, respectively. Data are represented as mean ± standard deviation.

To measure how much recombination gpII nicking induces, we determined the frequency of Ade^+^ recombinants both with and without gpII and/or its cleavage site (Fig. 5). A *his3*^+^ gene between the *ade6*^−^ heteroalleles allowed us to determine whether the Ade^+^ recombinants were from a gene conversion or deletion event (Fig. 5a). In preliminary experiments we found that pREP1-levels of gpII generated saturating amounts of recombinants, which made it difficult to obtain an accurate measure of recombination (data not shown). We therefore expressed gpII from the weaker *nmt* promoter in pREP81. The presence of gpII in the absence of its cleavage site, or a cleavage site without gpII, caused no increase in recombinants over spontaneous levels, whereas the two together resulted in ~15–21 fold increase in gene conversions and ~31–33 fold increase in deletions (Fig. 5b and see Supplementary Table S1). Very little difference was observed in the frequency of recombinants induced by a bottom strand nick compared to a top strand nick (Fig. 5b and see Supplementary Table S1), and therefore we chose to focus on strains with a bottom strand nick for the studies described below.

### Exo1 suppresses deletions and gene conversions caused by replication-associated DSBs

**Figure 6:**
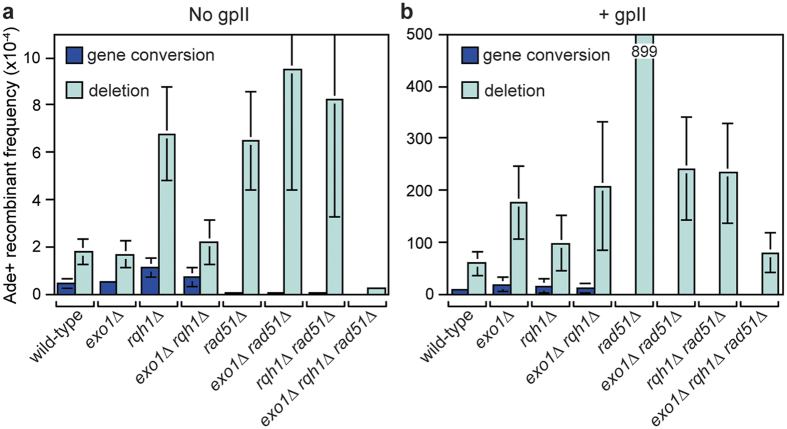
Spontaneous and gpII-induced direct repeat recombination in *exo1*∆, *rqh1*∆ and *rad51*∆ single, double and triple mutants. (**a**) Spontaneous Ade^+^ recombinant frequencies for strains MCW1159, MCW2209, MCW2070, MCW2202, MCW1894, MCW3494, MCW3492 and MCW3496 carrying pREP81 (see also Supplementary Table S1). (**b**) GpII-induced Ade^+^ recombinant frequencies for the same strains as in (**a**) but carrying pREP81-NLS-gpII instead of the empty pREP81 plasmid (see also Supplementary Table S1). Data are represented as mean ± standard deviation. Where a bar extends beyond the scale on the Y axis the value is indicated by the number in the bar.

We next determined what effect deleting *exo1* and *rqh1* has on both spontaneous and gpII-induced direct repeat recombination (Fig. 6 and see Supplementary Table S1). The frequency of spontaneous recombination was unaltered by deleting *exo1* (Fig. 6a and see Supplementary Table S1). In contrast, and consistent with previous data^37^, a *rqh1*∆ mutant exhibits a ~2 fold increase in gene conversions and ~4-fold increase in deletions (Fig. 6a and see Supplementary Table S1). Analysis of an *exo1*∆ *rqh1*∆ double mutant shows that this hyper-recombination is almost entirely dependent on *exo1* (Fig. 6a). Unlike spontaneous recombination, the overall frequency of gpII-induced recombinants increases by ~2.9-fold in an *exo1*∆ mutant (*P* < 0.001), which is more than the ~1.7-fold increase in a *rqh1*∆ mutant (*P* = 0.005) (Fig. 6b and see Supplementary Table S1). Moreover, an *exo1*∆ *rqh1*∆ double mutant exhibits a similar overall increase in recombinants as an *exo1*∆ single mutant (Fig. 6b and see Supplementary Table S1). These data suggest that either Exo1 and Rqh1 function in the same pathway for limiting gpII-induced recombinants or that Exo1 is required for the hyper-recombination in a *rqh1*∆ mutant.

### Exo1 and/or Rqh1 are required for the hyper-level of deletions in a *rad51*∆ mutant

In wild-type cells, direct repeat recombination is probably driven mainly by Rad51, giving rise to both gene conversions and deletions (see Supplementary Fig. S2)^38^. However, when Rad51 is absent recombination can be channelled into Rad52-dependent SSA, which generates solely deletions^38^. Consistently, we observed a loss of spontaneous gene conversions, together with a ~3.5 fold increase in deletions in a *rad51*∆ mutant (Fig. 6a and see Supplementary Table S1). Similar levels of deletions are observed in both *exo1*∆ *rad51*∆ and *rqh1*∆ *rad51*∆ double mutants, but an *exo1*∆ *rqh1*∆ *rad51*∆ triple mutant exhibits a ~24 fold reduction in deletions (Fig. 6a and see Supplementary Table S1). This finding is consistent with the need for either Exo1 or Rqh1 to catalyse the extensive DNA resection required for SSA of the *ade6*^−^ repeats that are ~5 kb apart, and tallies with previous findings in both budding and fission yeast^12^,^39^.

Similar to spontaneous recombination, Rad51 is also required for limiting deletions induced by gpII, but here the effect is even more striking with deletions increasing ~15 fold in a *rad51*∆ mutant compared to wild-type (Fig. 6b and see Supplementary Table S1). Unlike the spontaneous deletions in a *rad51*∆ mutant, the gpII-induced deletions are suppressed ~4 fold by deleting either *exo1* or *rqh1*, and loss of both results in a further ~3 fold reduction (Fig. 6b and see Supplementary Table S1). Together these data indicate that the accuracy of repair of broken replication forks is greatly enhanced by Rad51, which prevents repair being channelled into error-prone mechanisms such as SSA, which likely depend on extensive DNA resection catalysed by Exo1 and Rqh1. Additionally, the heightened level of gpII-induced deletions in an *exo1*∆ *rqh1*∆ double mutant is mostly suppressed by *rad51*∆, suggesting that long-range DNA resection helps promote the accuracy of Rad51-mediated recombination (i.e. accurate sister chromatid recombination instead of ectopic recombination between DNA repeats).

### UV-induced deletions and gene conversions depend on either Exo1 or Rqh1

**Figure 7:**
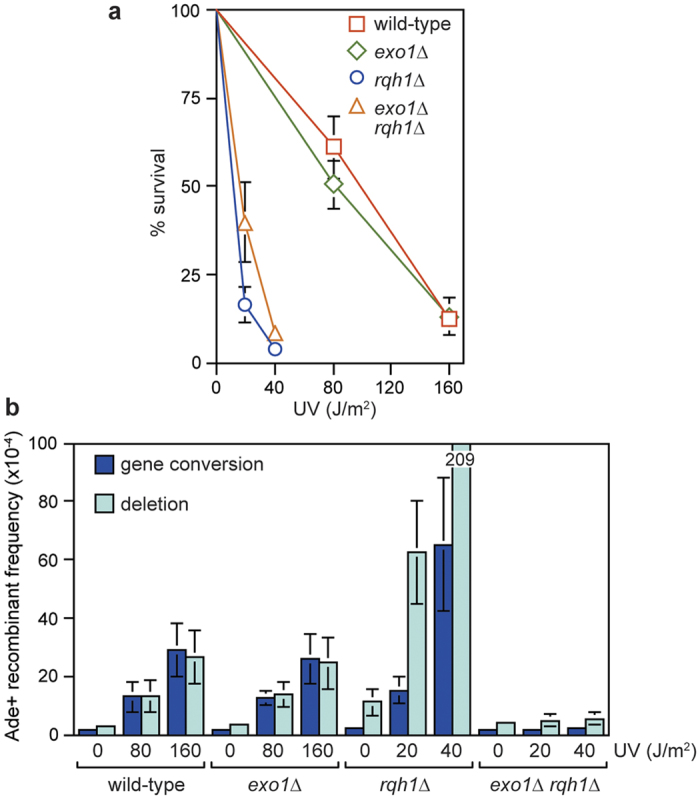
UV-induced direct repeat recombination in *exo1*∆ and *rqh1*∆ single and double mutants. **(a)** UV survival curves for strains MCW429, FO949, FO911 and FO1098. The error bars are the standard deviations about the mean. **(b)** UV-induced Ade^+^ recombinant frequencies for the same strains as in (**a**) (see also Supplementary Table S2). Data are represented as mean ± standard deviation. Where a bar extends beyond the scale on the Y axis the value is indicated by the number in the bar.

To see whether Exo1 and/or Rqh1 are required for promoting and/or suppressing the formation of recombinants induced by other replication fork perturbing lesions, we measured direct repeat recombination following UV exposure in *exo1*∆ and *rqh1*∆ single and double mutants (Fig. 7). As both *rqh1*∆ single and *exo1*∆ *rqh1*∆ double mutants are far more sensitive to UV than either wild type or an *exo1*∆ single mutant, we chose UV doses that gave similar percentages of cell survival across the four strains (Fig. 7a). Interestingly we observed that the hyper-sensitivity to UV of a *rqh1*∆ mutant was partially suppressed by deleting *exo1*. UV strongly induces the frequency of both gene conversion (~22 fold at 160 J/m^2^ UV) and deletions (~9 fold at 160 J/m^2^ UV) in a wild-type strain (Fig. 7b and see Supplementary Table S2). In contrast to what happens with gpII, the frequency of UV-induced recombinants is not affected by deleting *exo1*, whereas deleting *rqh1* results in a dramatic further increase in recombinants (~28 fold increase in gene conversions and ~19 fold increase in deletions at 40 J/m^2^ UV) (Fig. 7b and see Supplementary Table S2). Strikingly, in an *exo1*∆ *rqh1*∆ double mutant there was very little or no induction of recombinants by UV (Fig. 7b and see Supplementary Table S2). These data suggest that DNA resection is necessary for UV-induction of both gene conversions and deletions, including the very high level of recombinants in a *rqh1*∆ mutant.

### Exo1 promotes the formation of gene conversions and deletions induced by replication fork blockage at *RTS1*

**Figure 8:**
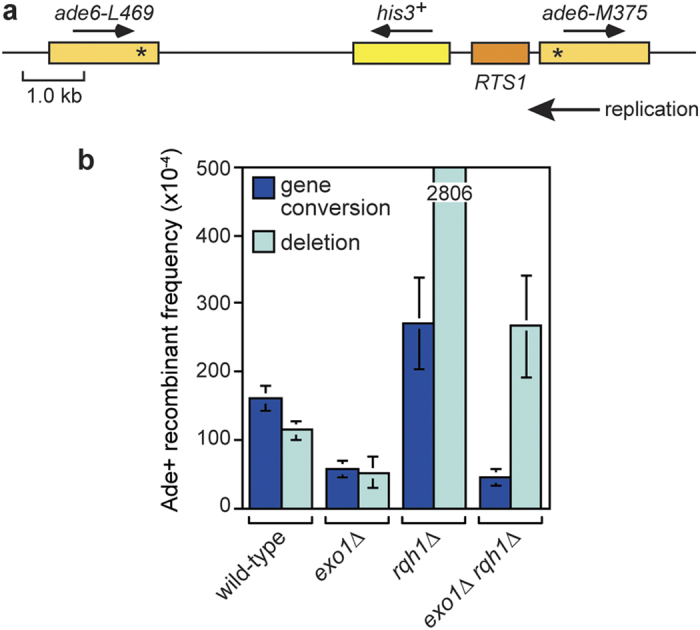
*RTS1*-induced direct repeat recombination in *exo1*∆ and *rqh1*∆ single and double mutants. **(a)** Schematic of the *ade6*^−^ direct repeat on chromosome 3 showing the position of *RTS1*. The distance between the *ade6*^−^ heteroalleles is ~6 kb. **(b)** *RTS1*-induced Ade^+^ recombinant frequencies for strains MCW4713, FO1742, MCW1447 and FO1762 (see also Supplementary Table S3). Data are represented as mean ± standard deviation. Where a bar extends beyond the scale on the Y axis the value is indicated by the number in the bar.

As a final comparison to gpII-induced recombination, we analysed what effect *exo1*∆ and *rqh1*∆ have on direct repeat recombination induced by the protein-DNA replication fork barrier *RTS1*. Replication fork blockage at *RTS1* results in fork collapse without DNA breakage, followed by the recruitment of recombination proteins within ~10 minutes that restart replication^35^,^40^. Using strains in which *RTS1* is positioned at the same site between the *ade6*^−^ heteroalleles as the gpII cleavage site (Fig. 8a), we found that loss of *exo1* results in a reduction in both gene conversions (~3 fold) and deletions (~2 fold) compared to wild-type (Fig. 8b and see Supplementary Table S3). As shown previously, and similar to spontaneous recombination, a *rqh1*∆ mutant exhibits a modest (~1.7 fold) increase in gene conversions and a large (~25 fold) increase in deletions (Fig. 8b and see Supplementary Table S3)^33^,^34^. Exo1 is required for most of the deletions (~90%) and gene conversions (~84%) in an *rqh1*∆ mutant (Fig. 8b and see Supplementary Table S3).

## Discussion

There are four main findings in this study: 1) Exo1 and Rqh1 play overlapping roles in the repair of replication-associated DSBs; 2) Depending on the context, Exo1 can either promote or limit direct repeat recombination; 3) In the absence of Exo1, Rqh1 promotes UV-induced direct repeat recombination; and 4) Rqh1, whilst playing a major role in limiting Exo1-dependent spontaneous, UV-induced, and *RTS1*-induced direct repeat recombination, has at best only a minor role in constraining recombination induced by a broken replication fork. These findings are discussed below.

Based on the known activities of Exo1 and Rqh1 in catalysing DNA resection, we suspect that the strong synergistic increase in sensitivity of an *exo1*∆ *rqh1*∆ double mutant, to conditions that generate SSBs, reflects an important role for long-range resection in repairing broken replication forks. Why IR-induced DSBs fail to elicit the same synergistic response is unclear. One possibility is that the importance of long-range resection is masked by another vital role that Rqh1 plays, such as the dissolution of double Holliday junctions^23^. Indeed, a *rqh1*∆ single mutant is already hypersensitive to IR, and further disablement of long-range resection may not appreciably add to its sensitivity. In fact in the case of UV-induced damage, loss of Exo1 appears to slightly suppress *rqh1*∆ sensitivity (Fig. 7A), which could be explained if long-range resection results in more Holliday junctions that have to be processed by Rqh1.

Alternatively, the method of delivery of IR might underlie the lack of synergistic enhancement in sensitivity in an *exo1*∆ *rqh1*∆ double mutant. In our experiments IR is given as an acute dose to an asynchronously growing culture and, therefore, ~90% of the cells experience the DNA damage outside of S-phase and likely repair it before replication starts. In contrast, CPT is delivered as a chronic dose and, therefore, every cell will be exposed to unrepaired SSBs during each S-phase. Interestingly, even in budding yeast, where an increase in sensitivity to IR is observed for an *exo1*∆ *sgs1*∆ double mutant^41^, the loss of long-range resection reduces the efficiency of gene conversion during DSB repair by only 30–50% in vegetative cells^12^,^13^. Why the repair of IR-induced DSBs, made predominantly outside of S-phase, might be less dependent on long-range resection than replication-associated DSBs is unclear. It has been proposed that one of the main benefits of long-range resection is to promote accurate recombinational repair of DSBs, which it does by providing a longer ssDNA tail for Rad51 to use for its homology search^13^. Without this resection only short ssDNA tails would be produced, and these would be more liable to undergo recombination with short repetitive DNA elements at ectopic sites. It is conceivable that the accuracy of recombination could be more critical when restarting replication than it is for repairing a DSB. The reason is that restarted replication can proceed until it encounters an opposing replication fork^35^,^42^ and, therefore, if initiated by strand invasion at an ectopic site, a genomic rearrangement will be inevitable. In contrast, DSB repair in G2 phase cells occurs mainly by SDSA, which is terminated when the invading strand is unwound and anneals to the other end of the break^3^. As such, SDSA would be less prone to generating major genome rearrangements, even when the initial strand invasion occurs at an ectopic site.

Whilst long-range resection might promote the fidelity of DSB repair, it could also encourage ectopic recombination by exposing repetitive DNA elements that reside further from a DSB or collapsed replication fork^13^ (see Supplementary Fig. S2). In our study we used direct repeats that are ~5 kb apart (or ~6 kb with *RTS1*) to measure the frequency of ectopic recombination. In the case of spontaneous direct repeat recombination, the loss of both Exo1 and Rqh1 has relatively little effect on the frequency of both gene conversions and deletions. However, the hyper-recombination seen in a *rqh1*∆ mutant depends on Exo1. The implication is that spontaneous direct repeat recombination is promoted by long-range resection but at the same time is limited by the action of Rqh1, possibly by means of its putative ability to disrupt D-loops or process double Holliday junctions^23^,^43^ (see Supplementary Fig. S2). The same appears to be true for *RTS1*-induced recombination, however, in this case, deletion of just *exo1* is sufficient to reduce the frequency of recombinants by ~50%, consistent with a previous study^44^. Moreover, no further reduction is seen in an *exo1*∆ *rqh1*∆ double mutant, suggesting that long-range resection is not essential for direct repeat recombination. However, it is possible that the true impact of losing long-range resection is masked by the loss of Rqh1’s anti-recombinogenic activity.

In contrast to spontaneous and *RTS1*-induced recombination, Exo1 appears to be needed to limit gpII-induced direct repeat recombination. As the gpII cleavage site is immediately adjacent to the *ade6-M375* allele in the direct repeat, short-range resection would be sufficient to expose the DNA repeat for binding and recombination by Rad51, whereas long-range resection would likely extend beyond the repeat into unique DNA sequence and thereby help promote accurate sister chromatid recombination. Alternatively, Exo1 could directly promote repair of a gpII-induced nick through its ability to initiate DNA degradation from a SSB^45^, and thereby prevent replication fork breakage and recombination from occurring. However, we think that this is unlikely given that members of the RAD2 nuclease family are impeded by chemical modification or protein attachment to the 5′ DNA end^46^. Therefore it is likely that gpII’s covalent attachment to the 5′ DNA end would block Exo1 from directly processing the SSB.

In the absence of Rad51, spontaneous recombination is shunted into the Rad52-dependent SSA pathway that generates deletions^38^. These deletions depend on either Rqh1 or Exo1, consistent with the proposal that they are formed by SSA, which has been shown to depend on long-range resection in budding yeast^12^. Similar to DSB repair by SSA in budding yeast, either Exo1 or Rqh1 is sufficient to deliver *rad51*∆ mutant levels of deletions when the DNA repeats are ~5 kb apart. However, the same is not true for the very high level of gpII-induced deletions in a *rad51*∆ mutant, which are reduced ~4-fold by deleting either *exo1* or *rqh1*. Why neither Exo1 nor Rqh1 is sufficient on its own to deliver the full *rad51*∆ mutant level of deletions under these conditions is unclear, but a similar lack of complete redundancy has been observed in budding yeast^12^.

Unlike spontaneous, gpII-induced and *RTS1*-induced recombination, UV-induced direct repeat recombination is abolished in an *exo1*∆ *rqh1*∆ double mutant, which suggests that DNA resection is required to convert UV-induced lesions into recombinogenic lesions. It has long been established that UV induces recombination, however the nature of the actual recombinogenic lesion is still debated^47^. The principle lesions induced by UV-C (~254 nm) are pyrimidine dimers, and in *S. pombe* these are removed either by nucleotide excision repair (NER) or by an alternative excision repair mechanism dependent on the Uve1 endonuclease^48^. In budding yeast UV lesions were thought to be recombinogenic only in actively dividing cells^49^, consistent with the notion that replication fork blockage by an unrepaired pyrimidine dimer, or fork breakage through encounter with a single-strand DNA gap made during excision repair, is responsible for the stimulus in recombination^50^. However, more recent work, studying allelic recombination in diploid budding yeast, has lead to the proposal that at high UV doses (≥15 J/m^2^) a significant portion of UV-induced recombinants stem from Exo1-dependent extension of two nearby single-strand gaps, made by NER on opposite DNA strands, so that they overlap and form a DSB^47^,^51^. Our finding that UV-induced direct repeat recombination in fission yeast depends on either Exo1 or Rqh1 could be explained by the same mechanism. However, there are other possibilities, for example studies in human, *Xenopus* and yeast indicate that activation of the checkpoint kinase ATR depends in part on its recruitment to ssDNA by its partner protein ATRIP interacting with the ssDNA binding protein RPA^52^,^53^. We have shown previously that the intra-S-phase checkpoint is essential for the production of UV-induced recombinants^54^, and therefore it is conceivable that DNA resection is needed to activate and/or maintain the checkpoint, which in turn promotes recombination.

Another finding from our study is that Rqh1 plays little or no role in constraining gpII-induced direct repeat recombination. A contrast with its prominent role in limiting *RTS1*-induced recombination, and the role of other RecQ-type helicases in suppressing recombination following replication fork perturbation^22^,^33^,^55^. One way that Rqh1 could selectively limit direct repeat recombination at a stalled fork, but not a broken fork, is by preventing the stalled fork from becoming broken^33^. Alternatively Rqh1 could modulate regression of the stalled replication fork. Fork regression involves re-annealing of the template DNA strands and extrusion of a new DNA arm formed by the annealing of the nascent strands, which can act as a recombinogenic substrate^56^. A common ability of RecQ-type helicases is catalysis of fork regression, and at least in the case of human RECQL1 the reverse reaction of fork restoration is also promoted^57^. In theory either fork regression or restoration could limit direct repeat recombination at *RTS1*. The former could extend the region behind the barrier that is bound by Rad51, thereby allowing DNA beyond the repeat to be used in the homology search, which in turn would promote more accurate sister chromatid recombination. Similarly, limiting fork regression by catalysing restoration could prevent the spread of Rad51 behind *RTS1* onto the DNA repeat, which in this case would also promote accurate sister chromatid recombination.

## Conclusion

We have highlighted how long-range resection may be especially important for the repair of broken replication forks in fission yeast and documented how it might promote faithful repair by limiting Rad51-mediated recombination between ectopic sites. We have also shown that Rqh1 limits Exo1-dependent ectopic recombination at stalled but not broken replication forks. In future work it will be important to determine whether the repair of replication-associated DSBs is truly more reliant on long-range resection than IR-induced DSBs, and exactly how Rqh1 limits direct repeat recombination.

## Methods

### Strains, plasmids and probes

*S. pombe* strains are listed in Supplementary Table S4. The gpII nickase site containing strains were made by targeted integration of BlpI linearised derivatives of pFOX2^58^ (see below) at *ade6-M375* in FO126 or FO1236. Southern blotting and diagnostic PCRs were used to determine that the linear plasmid had integrated at the correct site. The top strand nick site-containing derivative, pJS33, was constructed by inserting SmaI - SalI digested annealed oligonucleotides containing the nick site, oMW482 (5′-TTTAAACCCGGGCTCGAGGTTCTTTAATAGTGGACTCTTGTTCCAAACTGGAACAACAGTCGACTTTAAA-3′) and oMW483 (5′-TTTAAAGTCGACTGTTGTTCCAGTTTGGAACAAGAGTCCACTATTAAAGAACCTCGAGCCCGGGTTTAAA-3′), into the EcoRV - SalI sites of pFOX2. The bottom strand nick site-containing derivative, pJS34, was similarly constructed using oMW480 (5′-TTTAAAGTCGACCTCGAGGTTCTTTAATAGTGGACTCTTGTTCCAAACTGGAACAACACCCGGGTTTAAA-3′) and oMW481 (5′- TTTAAACCCGGGTGTTGTTCCAGTTTGGAACAAGAGTCCACTATTAAAGAACCTCGAGGTCGACTTTAAA-3′). pREP1-NLS-gpII was constructed by PCR amplification of the *gpII* gene from pK125^31^ using primers oMW409 (5′-TTTTTCCATATGGGAAGTCCTAAGAAGAAACGAAAGGTGATGATTGACAT GCTAGTTTTAC- 3′) and oMW410 (5′- TTGGATCCTTATGCGATTTTAAGAACTG- 3′), which were designed to introduce a nuclear localisation signal (NLS) and NdeI site at its 5′-end and a BamHI site at its 3′-end. The restriction sites were used to facilitate cloning into pREP1. pREP81-NLS-gpII was derived from pREP1-NLS-gpII by subcloning. All plasmids were sequenced to confirm that no mutations had been introduced during the cloning. Probes A and B have been described previously^33^, and probe E is a 915 bp BlpI - NdeI fragment from pFOX2.

### Media and genetic methods

Standard protocols were used for the growth and genetic manipulation of *S. pombe*^59^. The complete and minimal media were yeast extract with supplements (YES) and Edinburgh minimal medium plus 3.7 mg/ml sodium glutamate (EMMG) and appropriate amino acids (0.25 mg/ml), respectively. Ade^+^ recombinants were selected on YES lacking adenine and supplemented with 0.2 mg/ml guanine to prevent uptake of residual adenine. Crosses were performed on malt extract (ME) agar with the required amino acids (concentration 50 μg/ml). Determination of spore viability by random spore analysis has been described previously^27^.

### Spot assays

Cells growing exponentially in YES at 30 °C were harvested, washed, counted using a haemocytometer, and resuspended in water at a density of 1 × 10^7^ cells/ml. Suspensions were serially diluted in 10-fold steps to 1 × 10^3^ cells/ml, and 10 μl aliquots of each dilution were then spotted onto YES plates with and without genotoxins as indicated. For the spot assay in Fig. 2b, alternating dilutions of 2-fold and 5-fold were used between spots. For ultraviolet light (UV) and ionising radiation (IR), plates were irradiated using a Stratalinker (Stratagene) and a Cesium source, respectively, after spotting. Plates were photographed after 4–5 days incubation at 30 °C. All spot assays were repeated at least once to ensure that results were reproducible.

### *S. pombe* genomic DNA preparation and gel electrophoresis

Yeast cultures were grown in EMMG lacking leucine and thiamine at 30 °C, with aeration for 24 hours. Cells were then harvested, and genomic DNA prepared in 0.75% agarose plugs using a CHEF genomic DNA plug kit (Bio-Rad) according to the manufacturer’s instructions. DNA in plugs was digested over night at 37 °C with 40–100 units of restriction enzyme. Plugs were typically cut in half to allow loading onto neutral and alkaline gels in parallel. Neutral gels were 0.8% agarose and were run in 1× TBE at 60 V for ~16 hours. Alkaline gels were run as described^60^ using a 50 mM NaOH 2 mM EDTA buffer system. All gels were Southern blotted and probed with ^32^P-labelled probe as indicated. Blots were analysed by Phosphor Imaging using a Fuji FLA3000 and Image Gauge software.

### Recombination assays

Mitotic recombination was assayed using strains containing a non-tandem direct repeat of *ade6* heteroalleles flanking a functional *his3* gene. Spontaneous, UV, gpII- and *RTS1*-induced Ade^+^ recombinant frequencies were measured as described previously^58^. To select for plasmids and allow for expression of gpII, cells were grown on EMMG lacking leucine and thiamine for 6–7 days at 30 °C prior to assessing the frequency of Ade^+^ recombinants. Recombinant frequencies represent the mean value from at least 15 colonies for each strain, and for strains containing plasmids at least three independent transformants were tested. The meiotic recombination assay has been described previously^27^. The statistical significance of differences between recombinant frequencies was calculated with two-sample t-tests.

## Additional Information

**How to cite this article**: Osman, F. *et al*. The RecQ DNA helicase Rqh1 constrains Exonuclease 1-dependent recombination at stalled replication forks. *Sci. Rep.* **6**, 22837; doi: 10.1038/srep22837 (2016).

## Supplementary Material

Supplementary Information
