# Supplementary material for: The RecQ DNA helicase Rqh1 constrains Exonuclease 1-dependent recombination at stalled replication forks
Source: Sci Rep. 2016 Mar 9;6:22837. doi: 10.1038/srep22837 (PMC4783781; doi:10.1038/srep22837)
Supplement: Supplementary Information [file srep22837-s1.pdf]

The RecQ DNA helicase Rqh1 constrains Exonuclease 1-dependent recombination at stalled replication forks

Fekret Osman, Jong Sook Ahn, Alexander Lorenz and Matthew C. Whitby

Supplementary Table S1: GpII-induced direct repeat recombination

Genotype	Nick site[a]	gpII[b]	Strain number	Number of colonies analysed	Ade⁺ His⁺ recombinant frequency (x 10^{-4})[c]		Ade⁺ His⁻ recombinant frequency (x 10^{-4})[c]	
					Mean	*P* value[d]	Mean	*P* value[d]
wild-type	-	-	MCW39	15	0.38 (+/- 0.19)	-	1.30 (+/- 0.55)	-
wild-type	-	+	MCW39	15	0.53 (+/- 0.29)	0.32[e]	1.45 (+/- 0.71)	0.58[e]
wild-type	BS	-	MCW1159	18	0.47 (+/- 0.21)	0.25[e]	1.81 (+/- 0.54)	0.054[e]
wild-type	BS	+	MCW1159	18	7.25 (+/- 3.00)	<0.001[e]	59.9 (+/- 22.4)	<0.001[e]
wild-type	TS	-	JSA267	18	0.43 (+/- 0.31)	0.42[e]	1.65 (+/- 0.88)	0.10[e]
wild-type	TS	+	JSA267	18	9.06 (+/- 3.64)	<0.001[e]	51.5 (+/- 19.6)	<0.001[e]
*exo1*Δ	BS	-	MCW2209	17	0.52 (+/- 0.20)	0.46[f]	1.67 (+/- 0.58)	0.46[f]
*exo1*Δ	BS	+	MCW2209	18	18.5 (+/- 13.84)	0.003[g]	175.9 (+/- 71.1)	<0.001[g]
*rqh1*Δ	BS	-	MCW2070	18	1.13 (+/- 0.42)	<0.001[f]	6.77 (+/- 1.98)	<0.001[f]
*rqh1*Δ	BS	+	MCW2070	18	15.9 (+/- 12.6)	0.011[g]	98.0 (+/- 53.3)	0.008[g]
*exo1*Δ *rqh1*Δ	BS	-	MCW2202	18	0.72 (+/- 0.40)	0.027[f]	2.21 (+/- 0.95)	0.13[f]
*exo1*Δ *rqh1*Δ	BS	+	MCW2202	18	12.9 (+/- 9.33)	0.025[g]	207.6 (+/- 124.1)	<0.001[g]
*rad51*Δ	BS	-	MCW1894	18	0.03 (+/- 0.06)	<0.001[f]	6.46 (+/- 2.08)	<0.001[f]
*rad51*Δ	BS	+	MCW1894	18	0.12 (+/- 0.27)	<0.001[g]	899.2 (+/- 193.2)	<0.001[g]

*exo1*Δ *rad51*Δ	BS	-	MCW3494	17	0.05 (+/- 0.05)	<0.001[f]	9.53 (+/- 5.13)	<0.001[f]
*exo1*Δ *rad51*Δ	BS	+	MCW3494	18	0.14 (+/- 0.29)	<0.001[g]	240.6 (+/- 99.0)	<0.001[g]
*rqh1*Δ *rad51*Δ	BS	-	MCW3492	18	0.01 (+/- 0.02)	<0.001[f]	8.23 (+/- 4.98)	<0.001[f]
*rqh1*Δ *rad51*Δ	BS	+	MCW3492	18	0.08 (+/- 0.23)	<0.001[g]	233.5 (+/- 95.5)	<0.001[g]
*exo1*Δ *rqh1*Δ *rad51*Δ	BS	-	MCW3496	21	0.00 (+/- 0.01)	<0.001[f]	0.27 (+/- 0.17)	<0.001[f]
*exo1*Δ *rqh1*Δ *rad51*Δ	BS	+	MCW3496	18	0.01 (+/- 0.04)	<0.001[g]	80.0 (+/- 38.3)	0.069[g]

[a] Position of gpII cleavage site: BS (bottom strand = lagging template strand); TS (top strand = leading template strand).

[b] Strains are transformed with the empty pREP81 plasmid (-) or pREP81-NLS-gpII (+) that expresses gpII nickase.

[c] The values in parentheses are the standard deviations about the mean.

[d] *P* values are derived from independent-sample *t*-tests comparing the mean values as indicated.

[e] Compared to the equivalent mean recombinant frequency in wild-type without gpII and its cleavage site.

[f] Compared to the equivalent mean recombinant frequency in wild-type with a bottom strand cleavage site but without gpII.

[g] Compared to the equivalent mean recombinant frequency in wild-type with a bottom strand cleavage site and gpII.

Supplementary Table S2: UV-induced direct repeat recombination

Genotype	Strain number	Number of colonies analysed	UV dose (J/m^2)	% survival[a]	Ade^+ His^+ recombinant frequency (x 10^{-4})[a]		Ade^+ His^- recombinant frequency (x 10^{-4})[a]	
					Mean	*P* value[b]	Mean	*P* value[b]
wild-type	MCW429	21	0	100	1.34 (+/- 0.60)	-	3.04 (+/- 1.30)	-
			80	61.2 (+/- 8.8)	13.0 (+/- 5.2)	<0.001[c]	13.1 (+/- 5.5)	<0.001[c]
			160	12.6 (+/- 2.9)	29.2 (+/- 9.1)	<0.001[c]	26.5 (+/- 9.2)	<0.001[c]
*exo1*Δ	FO949	27	0	100	1.54 (+/- 0.60)	-	3.18 (+/- 0.85)	-
			80	50.6 (+/- 6.9)	12.4 (+/- 2.4)	<0.001[c]	13.6 (+/- 4.2)	<0.001[c]
			160	13.1 (+/- 5.2)	26.0 (+/- 8.8)	<0.001[c]	24.7 (+/- 8.8)	<0.001[c]
*rqh1*Δ	FO911	27	0	100	2.30 (+/- 1.34)	-	11.1 (+/- 4.5)	-
			20	16.4 (+/- 5.2)	15.2 (+/- 4.7)	<0.001[c]	62.6 (+/- 17.8)	<0.001[c]
			40	4.0 (+/- 1.1)	65.1 (+/- 22.9)	<0.001[c]	209.1 (+/- 59.9)	<0.001[c]
*exo1*Δ *rqh1*Δ	FO1098	27	0	100	1.45 (+/- 0.84)	-	3.83 (+/- 1.43)	-
			20	39.7 (+/- 11.3)	1.69 (+/- 1.05)	0.35[c]	4.76 (+/- 2.09)	0.06[c]
			40	8.5 (+/- 3.4)	2.33 (+/- 1.41)	0.008[c]	5.50 (+/- 2.02)	0.002[c]

[a] The values in parentheses are the standard deviations about the mean.
[b] *P* values are derived from paired *t*-tests comparing the mean values as indicated.
[c] Compared to the equivalent mean recombinant frequency for 0 J/m^2 UV.

Supplementary Table S3: *RTS1*-induced direct repeat recombination

Genotype	*RTS1*	Strain number	Number of colonies analysed	Ade+ His+ recombinant frequency (x 10^{-4})[a]		Ade+ His- recombinant frequency (x 10^{-4})[a]	
				Mean	*P* value[b]	Mean	*P* value[b]
wild-type	AO	MCW4713	18	161.4 (+/- 19.4)	-	114.4 (+/- 14.1)	-
*exo1*Δ	AO	FO1742	21	57.1 (+/- 11.6)	<0.001[c]	51.8 (+/- 23.3)	<0.001[c]
*rqh1*Δ	AO	MCW1447	16	270.4 (+/- 67.9)	<0.001[c]	2806.8 (+/- 1044.8)	<0.001[c]
*exo1*Δ *rqh1*Δ	AO	FO1762	21	44.4 (+/- 11.8)	<0.001[c]	266.4 (+/- 73.6)	<0.001[c]

[a] The values in parentheses are the standard deviations about the mean.
[b] *P* values are derived from independent-sample *t*-tests comparing the mean values as indicated.
[c] Compared to the equivalent mean recombinant frequency for wild-type.

Supplementary Table S4: List of *S. pombe* strains used in this study

Strain	Mating type	Genotype	Source
FO656	h^{+}	ura4-D18 his3-D1 leu1-32 arg3-D4	Lab strain
MCW1019	h^{+}	rqh1Δ::kanMx6 ura4-D18 his3-D1 leu1-32 arg3-D4	Lab strain
FO951	h^{+}	exo1Δ::$ura4^{+}$ ura4-D18 his3-D1 leu1-32 arg3-D4	Lab strain
FO1011	h^{+}	exo1Δ::$ura4^{+}$ rqh1Δ::kanMx6 ura4-D18 his3-D1 leu1-32 arg3-D4	This study
MCW2453	h^{+}	$rqh1^{K547A}$ ura4-D18 leu1-32	ECS218[a]
MCW2454	h^{+}	$rqh1^{K547R}$ ura4-D18 leu1-32	ECS220[a]
MCW4029	h^{-}	exo1Δ::$ura4^{+}$ $rqh1^{K547A}$ ura4-D18 his3-D1 leu1-32 arg3-D4	This study[a]
MCW4030	h^{-}	exo1Δ::$ura4^{+}$ $rqh1^{K547R}$ ura4-D18 his3-D1 leu1-32 arg3-D4	This study[a]
MCW3605	h^{-}	mat2,3Δ::$LEU2^{+}$ rqh1Δ::kanMx6 exo1Δ::$ura4^{+}$ ura4-D18 leu1-32 arg3-D4	This study[b]
MCW3664	h^{-}	mat2,3Δ::$LEU2^{+}$ ura4-D18 leu1-32 arg3-D4	This study[b]
MCW3665	h^{-}	mat2,3Δ::$LEU2^{+}$ exo1Δ::$ura4^{+}$ ura4-D18 leu1-32 arg3-D4	This study[b]
MCW3666	h^{-}	mat2,3Δ::$LEU2^{+}$ rqh1Δ::kanMx6 ura4-D18 leu1-32 arg3-D4	This study[b]
MCW3667	h^{-smt0}	mat2,3Δ::$LEU2^{+}$ ura4-D18 leu1-32 arg3-D4	This study[c]
MCW3668	h^{-smt0}	mat2,3Δ::$LEU2^{+}$ exo1Δ::$ura4^{+}$ ura4-D18 leu1-32 arg3-D4	This study[c]
MCW3669	h^{-smt0}	mat2,3Δ::$LEU2^{+}$ rqh1Δ::kanMx6 ura4-D18 leu1-32 arg3-D4	This study[c]
MCW3670	h^{-smt0}	mat2,3Δ::$LEU2^{+}$ rqh1Δ::kanMx6 exo1Δ::$ura4^{+}$ ura4-D18 leu1-32 arg3-D4	This study[c]
MCW39	h^{-}	ura4-D18 leu1-32 his3-D1 ade6-L469/pUC8/$his3^{+}$/ade6-M375	Lab strain
MCW1159	h^{-}	ura4-D18 leu1-32 his3-D1 ade6-L469/pUC8/$his3^{+}$/BS nick site/ade6-M375	This study
JSA267	h^{-}	ura4-D18 leu1-32 his3-D1 ade6-L469/pUC8/$his3^{+}$/TS nick site/ade6-M375	This study
MCW2391	h^{-}	rad50S ura4-D18 leu1-32 his3-D1 ade6-L469/pUC8/$his3^{+}$/BS nick site/ade6-M375	This study
MCW2393	h^{-}	rad50S ura4-D18 leu1-32 his3-D1 ade6-L469/pUC8/$his3^{+}$/TS nick site/ade6-M375	This study
MCW2746	h^{-}	rad50S cdc10-V50 ura4-D18 leu1-32 his3-D1 ade6-L469/pUC8/$his3^{+}$/BS nick site/ade6-M375	This study
MCW2070	h^{-}	rqh1Δ::kanMx6 ura4-D18 leu1-32 his3-D1 ade6-L469/pUC8/$his3^{+}$/BS nick site/ade6-M375	This study
MCW2202	h^{+}	rqh1Δ::kanMx6 exo1Δ::$ura4^{+}$ ura4-D18 leu1-32 his3-D1 ade6-L469/pUC8/$his3^{+}$/BS nick site/ade6-M375	This study
MCW2209	h^{+}	exo1Δ::$ura4^{+}$ ura4-D18 leu1-32 his3-D1 ade6-L469/pUC8/$his3^{+}$/BS nick site/ade6-M375	This study
MCW1894	h^{+}	rad51Δ::$ura4^{+}$ ura4-D18 leu1-32 his3-D1 ade6-L469/pUC8/$his3^{+}$/BS nick site/ade6-M375	This study
MCW3492	h^{+}	rad51Δ::$arg3^{+}$ rqh1Δ::kanMx6 ura4-D18 leu1-32 his3-D1 ade6-L469/pUC8/$his3^{+}$/BS nick site/ade6-M375	This study
MCW3494	h^{+}	rad51Δ::$arg3^{+}$ exo1Δ::$ura4^{+}$ ura4-D18 leu1-32 his3-D1 ade6-L469/pUC8/$his3^{+}$/BS nick site/ade6-M375	This study
MCW3496	h^{+}	rad51Δ::$arg3^{+}$ exo1Δ::$ura4^{+}$ rqh1Δ::kanMx6 ura4-D18 leu1-32 his3-D1 ade6-L469/pUC8/$his3^{+}$/BS nick site/ade6-M375	This study
MCW429	h^{+}	ura4-D18 leu1-32 his3-D1 arg3-D4 ade6-L469/pUC8/$his3^{+}$/ade6-M375	This study
FO911	h^{+}	rqh1Δ::kanMx6 ura4-D18 leu1-32 his3-D1 arg3-D4 ade6-L469/pUC8/$his3^{+}$/ade6-M375	This study
FO949	h^{+}	exo1Δ::$ura4^{+}$ ura4-D18 leu1-32 his3-D1 arg3-D4 ade6-L469/pUC8/$his3^{+}$/ade6-M375	This study
FO1098	h^{-}	exo1Δ::$ura4^{+}$ rqh1Δ::kanMx6 ura4-D18 leu1-32 his3-D1	This study

		arg3-D4 ade6-L469/pUC8/his3^{+}/ade6-M375	
MCW4713	h^{+}	*ade6-M375 int::pUC8/his3^{+}/RTS1-AO/ade6-L469 ura4-D18 his3-D1 leu1-32 arg3-D4*	This study
FO1742	h^{+}	*exo1Δ::ura4^{+} ade6-M375 int::pUC8/his3^{+}/RTS1-AO/ade6-L469 ura4-D18 his3-D1 leu1-32 arg3-D4*	This study
MCW1447	h^{+}	*rqh1Δ::kanMx6 ade6-M375 int::pUC8/his3^{+}/RTS1-AO/ade6-L469 ura4-D18 his3-D1 leu1-32 arg3-D4*	This study
FO1762	h^{+}	*exo1Δ::ura4^{+} rqh1Δ::kanMx6 ade6-M375 int::pUC8/his3^{+}/RTS1-AO/ade6-L469 ura4-D18 his3-D1 leu1-32 arg3-D4*	This study
ALP649	h^{+}	wild type	Lab strain
ALP688	h^{-smt0}	wild type	Lab strain
MCW3748	h^{+}	*exo1Δ::ura4^{+} ura4-D18*	This study
MCW3749	h^{-smt0}	*exo1Δ::ura4^{+} ura4-D18*	This study
MCW3387	h^{+}	*rqh1Δ::kanMx6*	(2)
MCW3388	h^{-smt0}	*rqh1Δ::kanMx6*	(2)
MCW4479	h^{+}	*exo1Δ::ura4^{+} rqh1Δ::kanMx6 ura4-D18*	This study
MCW4480	h^{-smt0}	*exo1Δ::ura4^{+} rqh1Δ::kanMx6 ura4-D18*	This study
MCW3202	h^{+}	*ura4^{+}-aim2 ade6-3083 ura4-D18 leu1-32 his3-D1*	(3)
MCW3200	h^{-smt0}	*his3^{+}-aim ade6-L469 ura4-D18 his3-D1 arg3-D4*	(3)
MCW4268	h^{-smt0}	*exo1Δ::kanMX6 his3^{+}-aim ade6-L469 ura4-D18 his3-D1 arg3-D4*	This study
MCW4269	h^{+}	*exo1Δ::kanMX6 ura4^{+}-aim2 ade6-3083 ura4-D18 leu1-32 his3-D1*	This study
MCW3385	h^{+}	*rqh1Δ::kanMX6 ura4^{+}-aim2 ade6-3083 ura4-D18 leu1-32 his3-D1*	(4)
MCW3384	h^{-smt0}	*rqh1Δ::kanMX6 his3^{+}-aim ade6-L469 ura4-D18 his3-D1 arg3-D4*	(4)
MCW4270	h^{-smt0}	*exo1Δ::kanMX6 rqh1Δ::natMX6 his3^{+}-aim ade6-L469 ura4-D18 his3-D1 arg3-D4*	This study
MCW4271	h^{+}	*exo1Δ::kanMX6 rqh1Δ::natMX6 ura4^{+}-aim2 ade6-3083 ura4-D18 leu1-32 his3-D1*	This study

[a] derived from ECS218/220, which were a gift from Shao-Win Wang
[b] derived from PB70 (1)
[c] derived from JZ108 (1)

1. Roseaulin, L., Yamada, Y., Tsutsui, Y., Russell, P., Iwasaki, H. and Arcangioli, B. (2008) Mus81 is essential for sister chromatid recombination at broken replication forks. *EMBO J.*, **27**, 1378-1387.

2. Lorenz, A., Osman, F., Sun, W., Nandi, S., Steinacher, R. and Whitby, M.C. (2012) The fission yeast FANCM ortholog directs non-crossover recombination during meiosis. *Science*, **336**, 1585–1588.

3. Lorenz, A., West, S.C. and Whitby, M.C. (2010) The human Holliday junction resolvase GEN1 rescues the meiotic phenotype of a *Schizosaccharomyces pombe* mus81 mutant. *Nucleic Acids Res.*, **38**, 1866–1873.

4. Lorenz, A., Mehats, A., Osman, F. and Whitby, M.C. (2014) Rad51/Dmc1 paralogs and mediators oppose DNA helicases to limit hybrid DNA formation and promote crossovers during meiotic recombination. *Nucleic Acids Res.*, **42**, 13723-13735.

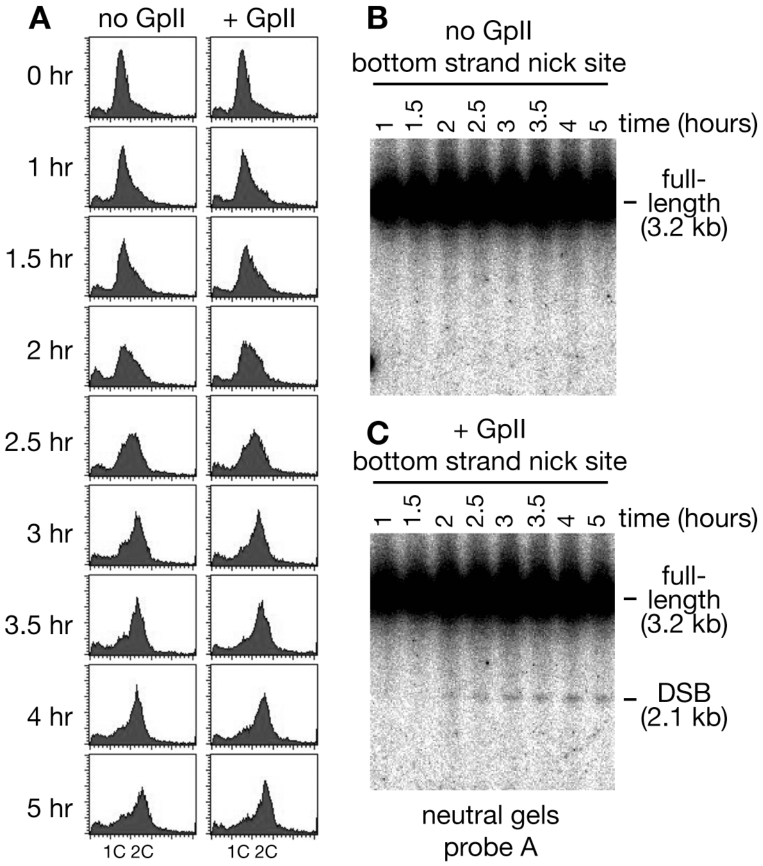

Supplementary Figure S1. GpII nick-induced DSB formation depends on passage through S-phase.

rad50s cdc10-V50 cells, with the bottom strand nick site and containing either pREP1 or pREP1-NLS-gpII, were grown at 28˚C for 20 hours in EMMG lacking histidine, leucine and thiamine to allow full de-repression of the *nmt* promoter, and therefore expression of NLS-gpII in cells containing pREP1-NLS-gpII. Cells were then transferred to fresh media and shifted to 34˚C to arrest them in G1. After 4 hours the cells were switched back to 28˚C and cell cycle progression and DSB formation monitored by flow cytometry (**A**) and neutral gel analysis (**B** and **C**), respectively. DNA was prepared in agarose plugs and digested with NdeI as in Figure 4C and D. Southern blots were probed with probe A (see Figure 4A).

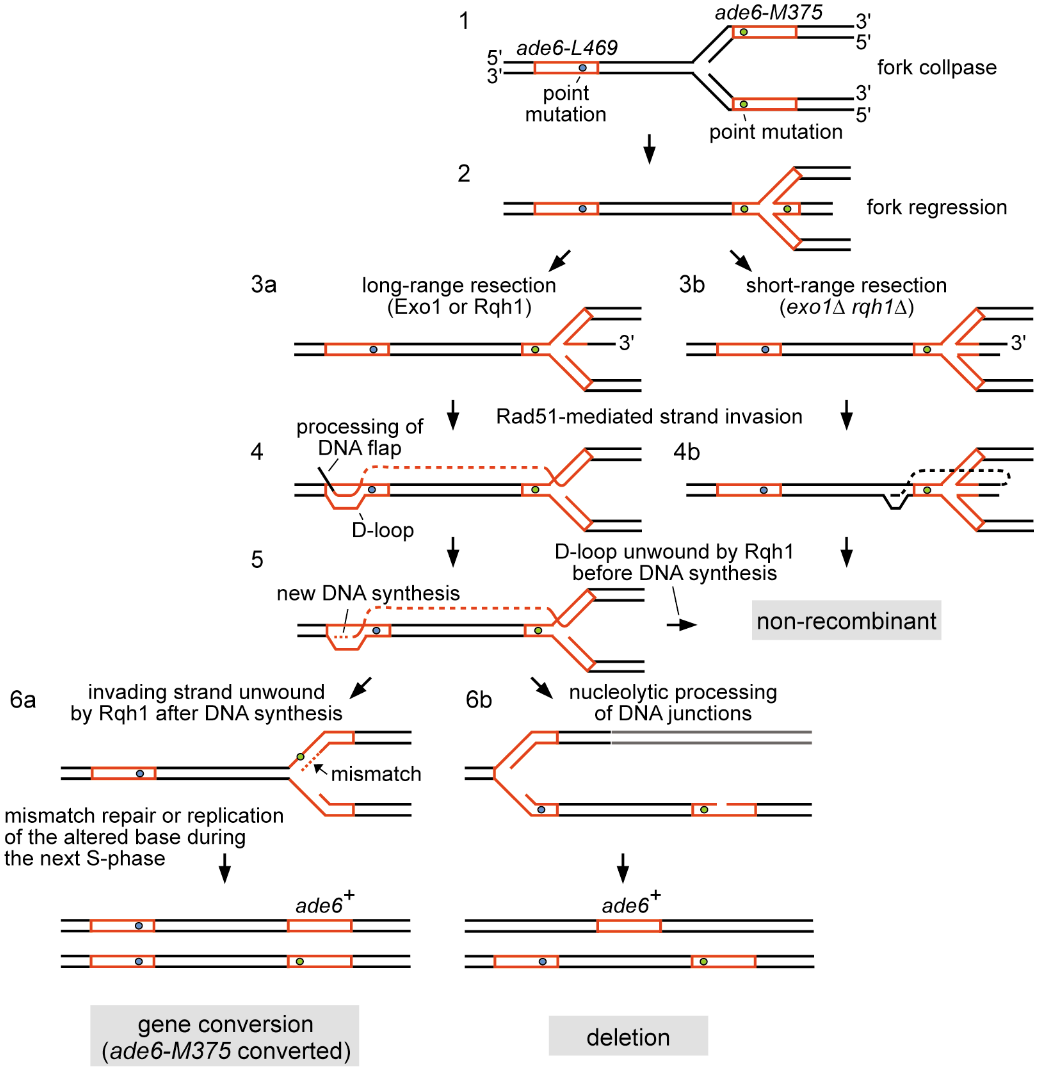

Supplementary Figure S2. Hypothetical model for how Exo1 and Rqh1 could influence the frequency of direct repeat recombination following replication fork collapse.
(**1**) Replication fork stalls and collapses between a direct repeat of *ade6*$^-$ heteroalleles. (**2**) Replication fork regression exposes a double-stranded DNA end. (**3a**) Long-range resection of the DNA end, catalysed by Exo1 or Rqh1, results in a 3' single-stranded tail consisting of non-repeated DNA (in black) and *ade6* repeated DNA (in red). (**4a**) Rad51 loads on to the 3' DNA tail and catalyses a search for homologous DNA. As the 3' tail consists of *ade6* DNA, Rad51 can use this to locate either *ade6* allele. In the diagram, Rad51 is shown catalysing DNA pairing and strand invasion with the *ade6-L469* allele. (**5**) Strand invasion, followed by processing of the heterologous DNA flap, exposes a 3' end from which new DNA synthesis can be primed. If processing of the flap results in removal of the *ade6-M375* point mutation, then the new DNA synthesis will result in the invading strand carrying just wild-type *ade6* genetic information. However, if Rqh1 unwinds the D-loop prior to DNA synthesis, it will result in a non-recombinant. (**6a**) Rqh1 unwinds the D-loop after a DNA polymerase has extended the 3' end, which ultimately leads to gene conversion of the *ade6-M375* allele. (**6b**) Cleavage of the two DNA junction points, by a structure-specific nuclease, such as Mus81-Eme1, results in an *ade6*$^+$ deletion-type recombinant. (**3b**) In the absence of Exo1 and Rqh1, the DNA end is subject to only short-range resection, which exposes only non-repeated DNA. (**4b**) Rad51-mediated strand invasion occurs only at non-repeated DNA and, therefore, does not generate an *ade6*$^+$ recombinant.
